# Isolated Scaphoid Dislocation Secondary to Pseudogout Arthritis: A Case Report

**DOI:** 10.7759/cureus.80651

**Published:** 2025-03-16

**Authors:** Wojciech K Dzieza, Marco A Foreman, Persis D Desai, Morad Chughtai, Anton Khlopas, Jongmin Kim

**Affiliations:** 1 Department of Orthopedic Surgery and Sports Medicine, College of Medicine, University of Florida, Gainesville, USA; 2 College of Medicine, University of Florida, Gainesville, USA; 3 Department of Hand, Upper Extremity, and Microvascular Surgery, Florida Orthopaedic Associates, DeLand, USA; 4 Department of Orthopedic Surgery, Henry Ford Health System, Wyandotte, USA

**Keywords:** atraumatic scaphoid dislocation, carpus arthropathy, crystal arthropathy, pseudogout, treatment of scaphoid dislocation

## Abstract

A 69-year-old male laborer with a prolonged history of untreated gouty arthritis in the wrist with an atraumatic isolated scaphoid dislocation requiring urgent salvage procedure in the form of proximal row carpectomy. Few isolated scaphoid dislocations have been reported in the literature. They usually are a result of high-energy mechanisms. To our knowledge, no atraumatic, isolated scaphoid dislocations with underlying pseudogout have been described in the literature. This report describes one such case and brings attention to the importance of treatment of inflammatory arthropathies in the carpus due to its effect on the integrity of the carpus and the scaphoid in particular.

## Introduction

Isolated scaphoid dislocations are a remarkably rare occurrence. There is a paucity of published literature on this subject, with only 55 reported cases between 1903 and 2020 [1]. Given the complex ligamentous support surrounding the scaphoid in the carpus, high-energy mechanisms are typically required for this event to occur [2]. Motor vehicle accidents (MVAs) are the most frequently described cause throughout the literature [1,3,4]. This injury is exceptionally uncommon considering the significant amount of energy required to displace the scaphoid from its anatomic position, more commonly resulting in fractures to the radial styloid or waist of the scaphoid [5]. In theory, however, factors that weaken the structural integrity of the scaphoid within the carpus could precipitate such an isolated injury.

Inflammatory conditions such as gout and calcium pyrophosphate deposition disease (CPPD) are two crystal arthropathies involving synovial and periarticular tissues that may occur in the distal upper extremity [6]. Pathologically, the destruction of interosseous soft tissues caused by crystalline deposition has the potential to lead to alteration of the native biomechanics of the wrist [7]. Poorly managed crystal arthropathies pose potential etiologies for carpal instability via compromised ligamentous scaffolding in conjunction with bony degeneration. Crystal arthropathy often causes scapholunate ligament disruption; however, this is the first report of an acute, atraumatic isolated scaphoid dislocation secondary to underlying CPPD [8].

This report describes a case of a scaphoid dislocation with a low-energy mechanism of injury in the setting of degenerative changes in the carpus caused by a combination of osteoarthritis and CPPD. The rare occurrence of isolated scaphoid dislocations, in general, as well as the unusual mechanism of injury in this particular case, prompted a formal report.

## Case presentation

A 69-year-old man presented with right wrist pain after experiencing a snapping sensation while mowing his lawn. He had struggled with chronic bilateral wrist pain for over 10 years. The patient has been followed by rheumatology for CPPD disease and was intermittently treated with colchicine. 

**Figure 1:**
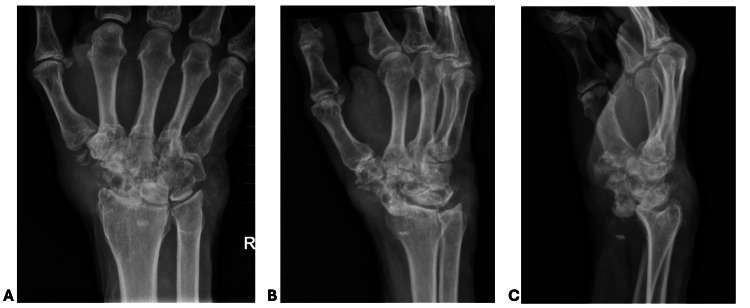
Injury radiographs of the right wrist AP: anteroposterior AP (A) and oblique X-ray (B) of the right wrist with extensive erosive changes throughout the carpus and soft tissue swelling surrounding the wrist. Lateral x-ray (C) demonstrating a frank volar scaphoid dislocation. Metacarpophalangeal (MCP) joints appear well-preserved in all projections

On examination, the patient was noted to have exquisite tenderness to palpation over the wrist, with prominent deformity and swelling along the radial aspect. The range of motion was limited. His distal extremity was otherwise well-perfused with grossly intact motor and sensory function. Radiographs demonstrated a frank volar scaphoid dislocation in the setting of erosive arthropathy throughout the carpus (Figure 1). The patient was initially splinted with a plan for wrist arthrodesis at a later date but returned to the clinic due to a new onset of paresthesias in the median nerve distribution and worsening pain. He was then taken to the operating room for an urgent operative treatment the same day.

A volar incision was first made directly over the scaphoid which was palpable around the flexor carpi radialis (FCR) tendon. Next, a dorsal approach through the third extensor compartment was used to enter the wrist capsule. The posterior interosseous nerve branch was identified, and a neurectomy was performed. Upon opening the capsule, brown, thick, purulent-like material was encountered. A proximal row carpectomy (PRC) and radial styloidectomy were performed. A dorsal wrist capsule was used to perform a capsular interposition around the capitate. The patient was placed in a well-padded volar splint and admitted for intravenous antibiotics given intraoperative findings and the possibility of an underlying superimposed infection.

**Figure 2:**
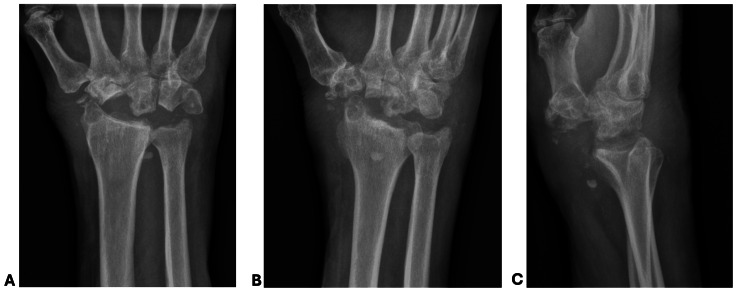
Postoperative radiographs of the right wrist at four weeks AP: anteroposterior Right wrist X-rays including AP (A), oblique (B), and lateral (C) projections with postoperative changes following proximal row carpectomy. No radiographic evidence of progressive erosive changes in the remaining carpal bones

The patient underwent intravenous antibiotic treatment for two weeks which was discontinued once intraoperative cultures were finalized and did not have any fungal, bacterial, or acid-fast bacilli growth. Synovial tissue was found to have CPPD deposits. At his four-week postoperative follow-up, his pain was improving, as was his range of motion (Figure 2). Five months following surgery, he was pleased with his regained range of motion and strength, with a grip strength of 35 lbs, measured using the Jamar Hand Dynamometer (JLW Instruments, Chicago, IL).

## Discussion

As described, isolated scaphoid dislocations are rare and often secondary to traumatic, high-energy injuries such as MVAs and those involving heavy machinery. In contrast to our case, the mechanism of injury often involves forced supination with hyperextension and axial loading. Comparatively, perilunate dislocations also arise from a common etiologic force. However, scaphoid dislocations are exceptionally rare due to additional volar stability provided by the radioscaphoid, radioscaphocapitate, and long radiolunate ligaments [9]. Consequently, diagnosis may be delayed or missed due to the rarity of this injury. Once the diagnosis has been made, treatment options include closed or open reduction with internal fixation and ligamentous reconstruction. Arthroscopy-assisted reduction is another potential treatment option which is less invasive and may theoretically preserve the blood supply [10]. Historically, a successful closed reduction has been the standard treatment option, but more recently definitive treatment has turned to open reduction with internal fixation and ligamentous reconstruction [1,11]. Complications from isolated scaphoid dislocations include degenerative joint instability and avascular necrosis [12].

In the context of nontraumatic injury to the carpus, solitary scaphoid dislocation is the scarcest sequelae. Presently, there are two known case reports of low-energy, isolated scaphoid dislocation secondary to scapholunate ligament disruption: one following routine dorsiflexion in a suspected Marfan syndrome patient and the other secondary to gout [8,11]. Thus, we present the first observed case of isolated scaphoid dislocation resulting from erosive arthropathy due to CPPD. A well-described possible consequence of untreated CPPD, known as scapholunate advanced collapse (SLAC), is an atraumatic pattern of wrist arthritis characterized by progressive deformity and instability of the radiocarpal and mid-carpal joints [13]. This condition presents with worsening pain, dorsoradial swelling, and an increasingly limited functional wrist arc. In conjunction with history and physical exam, SLAC is diagnosed with plain and advanced imaging. Similar to the preoperative radiographs of the present case, this arthropathy typically demonstrates erosive basal joint arthritis and concomitant chondrocalcinosis [13].

Conservative management of SLAC may include wrist immobilization, nonsteroidal anti-inflammatory medications, and intraarticular corticosteroid injections. As done in this case, when recalcitrant to nonoperative treatment, single and multistep procedures are often combined, ranging from radial styloidectomy, partial or total wrist arthrodesis, PRC, and neurectomy. According to recent literature, the subjective patient and functional outcomes of techniques such as PRC and four-corner arthrodesis (FCA) in treating SLAC are therapeutically satisfactory and comparable [14]. However, careful consideration should be practiced in younger patient populations due to higher rates of revision and secondary osteoarthritis in FCA compared to PRC in long-term studies [15].

Given the unusual mechanism of injury and the severity of the underlying degenerative and erosive process in the wrist of the patient in the presented case, the ultimate cause could be attributed to years of poorly controlled CPPD arthropathy in the wrist. Infection was ruled out with intraoperative cultures. Additionally, given the degeneration in the carpus, any surgical attempt at salvaging the scaphoid would be futile, making a PRC the most appropriate operative treatment option for this patient. While the erosive and degenerative changes in the carpus in the setting of crystal arthropathy occur over an extended period of time, the presented case highlights the importance of medical treatment and raises elective surgical treatment to question in an attempt to prevent a rare but potentially disabling consequence.

## Conclusions

Isolated scaphoid dislocations are rare and cannot be missed. Left untreated, they may lead to significantly impaired function of the wrist and hand. While scaphoid dislocations are most often caused by high-energy trauma, chronic underlying degeneration of the carpus due to conditions such as pseudogout may predispose patients to this pathology in nontrauma settings. Being mindful of the long-term consequences and potential sequelae of crystal arthropathies such as atraumatic scaphoid dislocation requiring urgent surgical treatment may better guide practitioners when treating affected patients.

## References

[1] Amundsen A, Bishop SN, Moran SL (2020). Isolated scaphoid dislocation: a case report and review of the literature. J Wrist Surg.

[2] Chniti I, Saybi F, Mahmoud AB, Mansi Z, Fradj AB, Rbai H (2023). Isolated partial carpal scaphoid dislocation: a case report. EAS J Orthop Physiother.

[3] Chloros GD, Themistocleous GS, Zagoreos NP, Korres DS, Efstathopoulos DG, Soucacos PN (2006). Isolated dislocation of the scaphoid. Arch Orthop Trauma Surg.

[4] Akinci M, Yildirim AO, Kati YA (2012). Late-presenting, isolated, complete radial dislocations of the scaphoid treated with the Szabo technique. J Hand Surg Eur Vol.

[5] Connell MC, Dyson RP (1955). Dislocation of the carpal scaphoid: report of a case. J Bone Joint Surg Br.

[6] Zamora EA, Naik R (2023). Calcium pyrophosphate deposition disease. https://www.ncbi.nlm.nih.gov/books/NBK540151/.

[7] Doherty W, Lovallo JL (1993). Scapholunate advanced collapse pattern of arthritis in calcium pyrophosphate deposition disease of the wrist. The. Journal of Hand Surgery.

[8] Lee YH, Tan HW, Lee HC (2008). Wrist gouty arthritis presenting as scaphoid erosions with scapholunate ligament disruption. Singapore Med J.

[9] Nathan R, Blatt G (2000). Rotary subluxation of the scaphoid: revisited. Hand Clin.

[10] Le Mapihan M, Dahan E, Bourcheix LM, Hardy A (2022). Arthroscopic preservation of the scaphoid's blood supply during nonunion treatment. J Hand Surg Eur Vol.

[11] Moy A, Song EY, Wallace SJ, Teixeira RM, Low YC, Weiss LE (2021). Isolated scaphoid dislocation from low-energy wrist trauma. J Hand Surg Glob Online.

[12] Amaravati RS, Saji M, Rajagopal H, Gururaj Gururaj (2009). Neglected dorsal dislocation of the scaphoid. Indian J Orthop.

[13] Shah CM, Stern PJ (2013). Scapholunate advanced collapse (SLAC) and scaphoid nonunion advanced collapse (SNAC) wrist arthritis. Curr Rev Musculoskelet Med.

[14] Reyniers P, van Beek N, De Schrijver F, Goeminne S (2023). Proximal row carpectomy versus four-corner arthrodesis in the treatment of SLAC and SNAC wrist: meta-analysis and literature review. Hand Surg Rehabil.

[15] Williams JB, Weiner H, Tyser AR (2018). Long-term outcome and secondary operations after proximal row carpectomy or four-corner arthrodesis. J Wrist Surg.

